# ECMO during the COVID-19 pandemic: When is it justified?

**DOI:** 10.1186/s13054-020-03386-4

**Published:** 2020-11-19

**Authors:** Silver Heinsar, Giles J. Peek, John F. Fraser

**Affiliations:** 1 Critical Care Research Group (CCRG), The Prince Charles Hospital, Chermside, Brisbane, QLD Australia; 2 Faculty of Medicine, University of Queensland, Brisbane, QLD Australia; 3 St Andrews War Memorial Hospital, Brisbane, QLD Australia; 4 Shand’s Congenital Heart Center, University of Florida, Gainesville, FL USA

## Background

**Fig. 1:**
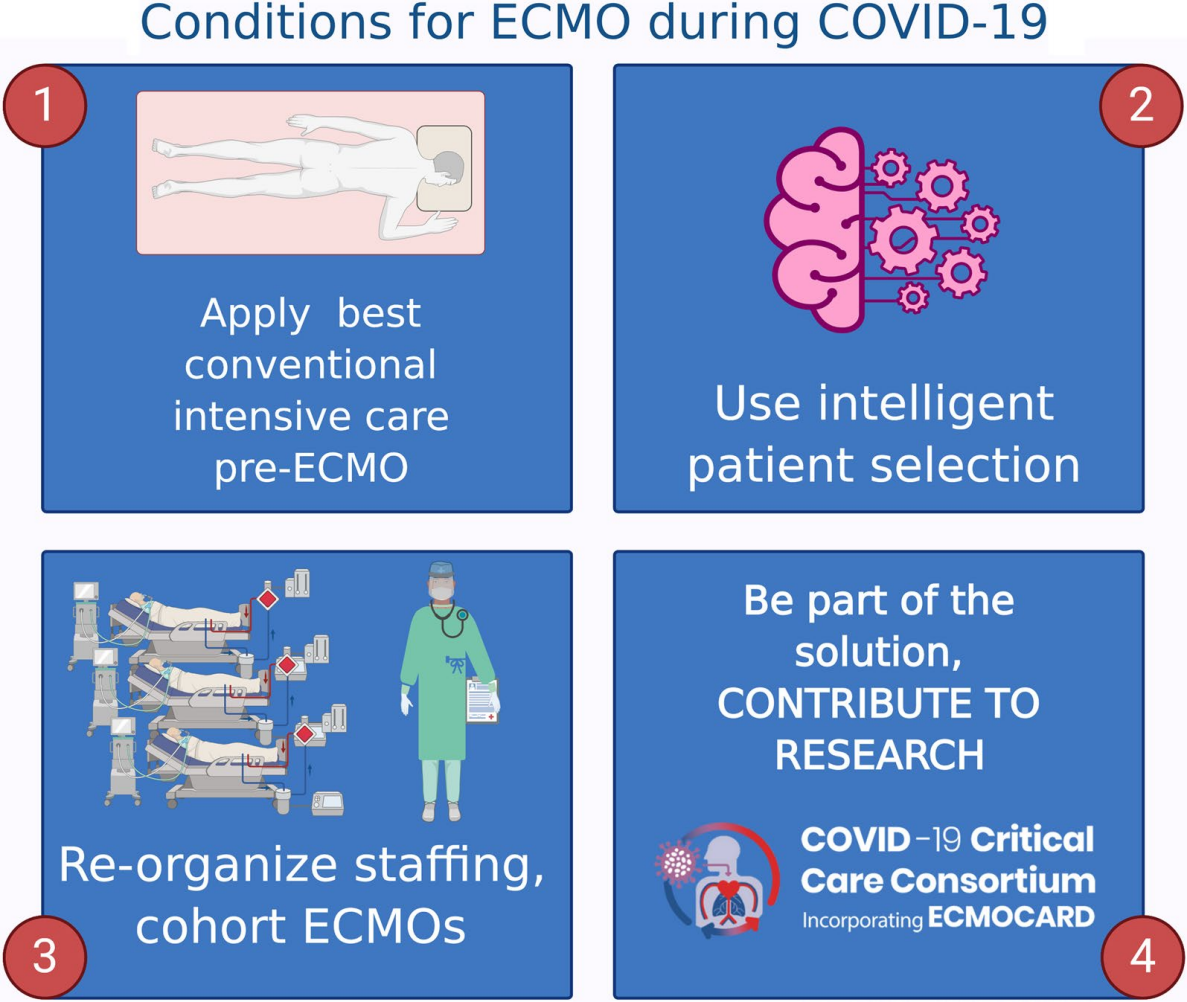
Conditions for ECMO during pandemic situations. Everything starts with providing best conventional intensive care measures prior to ECMO. Careful filtering of patients, which can be facilitated with AI and machine learning support is critical to achieve good outcomes. Resource allocation can preserve the quality of care within the unit. And finally, experience needs to be translated to evidence and contributing to research is the way to make ECMO count

Abrams et al. recently introduced their view of extracorporeal membrane oxygenation (ECMO) utilisation during the COVID-19 pandemic as a burden-based approach, highlighting that surge conditions may result in decreased utilization of ECMO, as resources must be carefully managed to ensure an acceptable level of care in all patients [1]. Whilst case numbers may overwhelm some health care systems, thus making ECMO seem less attractive, we are offering an extended viewpoint where ECMO may be justified if systems are optimised to proceed without compromising the overall delivery of intensive care (Fig. 1).

The objective of this commentary is to provide general guidance, to expand on previously described suggestions by Abrams et al., on ECMO use during pandemic situations, especially while treating the critically ill patients with COVID-19.

## Conditions allowing for judicious use of ECMO

### Firstly, apply the best conventional intensive care before moving to ECMO.

ECMO is a complex supportive treatment with inherent complications and significant economic implications. ECMO should only be considered when proven effective and relatively inexpensive measures such as proning, neuromuscular blockade and lung-protective ventilation have been tried without success. Omitting these steps without a valid reason prior to ECMO commencement is unjustified and a disservice to the global ECMO community.

### Secondly, pick the right patients for the right treatment.

The staggering contrast in outcomes of currently published ECMO reports amongst all COVID-19 patients tells a tale of different use of the same technology. The heterogeneity of COVID-19 patients with acute respiratory distress syndrome (ARDS) has been hypothesized by numerous authors, whilst no agreement on the existence and nature of possible sub-phenotypes exists [2, 3]. Furthermore, knowledge on COVID-19 atypical manifestations, such as predisposition to intrapulmonary thrombosis, right ventricular failure and the compounded immunologic insult by both COVID-19 infection and the extracorporeal circuit is yet to be fully explored. As the world is still waiting for comparative analyses of COVID-19 ECMO patients, intelligent ECMO case selection using the available evidence-base is essential to facilitate each patients’ recovery potential.

Considering the currently progressing global pandemic, we propose the use of advanced prediction models, which take into account regional differences, to complement expert opinions and various preliminary algorithms. Artificial intelligence (AI) and machine learning (ML) are well suited to analysing large volumes of complex, heterogenous data in order to guide the life-critical decisions intensivists are facing as the pandemic evolves [4].

As the global community is entering another wave of COVID-19, it’s inevitable the demand for ECMO will exceed its supply. In case of multiple candidates with appropriate indications, ICUs will need to implement triage to select the most deserving candidates, with considerations including but not isolated to age, comorbidities and days ventilated.

### Thirdly, safely utilise fewer specialized ECMO staff for more patients.

Resource allocation is the paramount in critical care during pandemic conditions. Cohorting ECMO patients and changing staffing ratios may facilitate satisfactory management of other ICU patients whilst not jeopardizing the general standard of care. The adoption of a allied health and nurse-driven program to ambulate patients on VV ECMO has been shown safe and may reduce other complications associated with immobility [5]. Furthermore, utilizing perfusionists as “ECMO specialists” can be done safely and could permit an expansive growth of ECMO in times of surge conditions [6]. Procedural management of ECMO must remain pragmatic and standardized to ensure staff burn out is prevented. Importantly, high-fidelity simulation programs are recommended as they reduce ECMO management time and improve teamwork [7].

### And finally, be part of the solution: contribute to research

Although well-conducted randomized controlled trials (RCTs) represent the best method of determining the efficacy of medical interventions, the eligibility criteria proposed by RCTs can make them unrepresentative of a wide population of patients. In addition, they are costly and time-consuming, particularly for medical devices such as ECMO. Factors such as low enrolment rate and crossovers also contribute to the impracticality of conducting large and timely RCTs of ECMO in critically ill patients, limiting the feasibility of applying this gold standard [8, 9]. Furthermore, a recently published meta-analysis combining 429 patients with ARDS from two large RCTs (CESAR and EOLIA) found significantly lower mortality in those treated with ECMO compared to conventional management, highlighting that additional efficacy trials in ECMO for ARDS are unnecessary [10]. Due to inappropriateness of a new RCT comparing different ECMO technologies, we recommend the use of modern causal inference methods, such as propensity matched pair analysis, to scale down the effect of confounding [11, 12]. Registries and consortiums collecting observational data on COVID-19 related ECMO are paramount to synthesize supportive evidence from geographically fragmented data, as ECMO remains a last resort measure, carefully selected for the sickest of the sick. Numerous large-scale registries collecting ECMO data in COVID-19 patients exist [13–15]. As clinical researchers, we urge all centres with capacity to perform ECMO support during this pandemic to find the most suitable and feasible way to collect and report their clinical data for maximal utilisation of clinically generated experience.

## Conclusion

In conclusion, whilst health care facilities must balance their intensive care resources with the fluctuations of patient volume as described by Abrams and colleagues, it’s imperative to ensure they’ve adhered to the highest standard of care prior to ECMO, used innovative and evidence-based solutions to decide who goes on ECMO, allocate specialized staff for those on ECMO to preserve the standard of care within the unit, and ensure their work gets translated to science and innovation by joining promising research endeavours in this field.

## Data Availability

Not applicable.
